# Perioperative Nutritional and Metabolic Factors Affecting Surgical Outcomes in Head and Neck Cancer Free Flap Reconstruction: A Comprehensive Review

**DOI:** 10.3390/jcm14113679

**Published:** 2025-05-23

**Authors:** Andrzej Jaxa-Kwiatkowski, Marek Jaxa-Kwiatkowski, Katarzyna Jaxa-Kwiatkowska, Hanna Gerber, Marcin Kubiak, Lidia Łysenko

**Affiliations:** 1 Clinical Department of Maxillofacial Surgery, Faculty of Dentistry, Wroclaw Medical University, 50-367 Wrocław, Poland; hanna.gerber@umw.edu.pl (H.G.); marcin.kubiak@umw.edu.pl (M.K.); 2 Department of Maxillofacial Surgery, Heliodor Swiecicki Clinical Hospital in Poznan, 60-355 Poznan, Poland; marekjaxa@interia.pl; 3 Division of Anatomy Part of the Department of Human Morphology and Embryology, Faculty of Medicine, Wroclaw Medical University, 50-367 Wrocław, Poland; katarzyna.staszak@umw.edu.pl; 4 Department and Clinic of Anaesthesiology and Intensive Care, Faculty of Medicine, Wroclaw Medical University, 50-367 Wrocław, Poland; lidia.lysenko@umw.edu.pl

**Keywords:** BMI, head and neck cancer, reconstructive surgery, oncology, nutrition

## Abstract

Head and neck cancer (HNC) remains a major global health issue. It is closely linked to smoking, alcohol use, and HPV infection. Nutritional and metabolic factors significantly influence surgical outcomes in these patients, especially when undergoing extensive resections and microsurgical free flap reconstruction. This comprehensive review aims to evaluate how perioperative nutritional status—particularly body mass index (BMI), serum albumin and prealbumin levels, and enteral vs. oral feeding strategies—affects complication rates, wound healing, surgical duration, and overall recovery. Poor nutritional status is associated with increased complication rates, prolonged surgery, impaired wound healing, and higher perioperative mortality. Both high and low BMI negatively impact surgical outcomes. Obesity is linked to protracted surgery and increased blood loss, while underweight patients have higher perioperative mortality. Optimizing perioperative nutrition is important for improving surgical outcomes in HNC patients. A multidisciplinary approach is necessary to tailor nutritional support and enhance recovery. Further research should focus on long-term weight management strategies and identifying biomarkers predictive of surgical success.

## 1. Introduction

Head and neck cancer (HNC) includes malignancies of the lip, oral cavity, oropharynx, hypopharynx, and larynx (International Classification of Diseases (ICD)-10 codes C00–C14, C30–C32, and C76). According to the International Agency for Research on Cancer, HNC is the seventh most common cancer globally [1]. Global Cancer Statistics reported approximately 890,000 new HNC cases and 450,000 deaths worldwide in 2020 [2]. Squamous cell carcinoma represents approximately 90% of HNC histological results [1]. The incidence rate in the United States is three times greater for men than for women, primarily affecting patients in their sixth decade [3]. In Poland, the estimated gender-related ratio is even higher at 9.6:1.8 (men/women). The highest incidence of lip cancer in Poland was noted in the Kielce Registry, whereas oral cavity cancers (ICD-10 codes C03–06) were more common in the Lower Silesia and Podkarpackie registries [4]. According to the 2020 Polish National Cancer Registry, 2792 new cases of oral cavity and oropharyngeal cancer (ICD-10 codes C00–C14) and 2253 deaths due to this cancer site were recorded [5]. The incidence and 5-year survival rates of HNC vary across countries; worldwide, the highest incidence is observed in India, where HNCs rank second in terms of both incidence and mortality rates, closely behind breast cancer [6]. Growing evidence suggests that perioperative outcomes in HNC patients are significantly influenced by body mass index (BMI), nutritional status, and metabolic factors. These elements are increasingly recognized as critical in optimizing surgical planning, wound healing, and overall recovery.

Globally, the major risk factors for HNCs are tobacco smoking and alcohol consumption. Combined exposure has a multiplicative effect compared to individuals who neither drink nor smoke [7]. In 2012, the number of HNC cases worldwide was less than 700,000; however, over the last eight years, a more than 27% increase has been observed [8,9]. The literature suggests that the annual incidence of HNCs is expected to increase further by 30–34% by 2030 due to growing trends of alcohol and tobacco use, the chewing of the carcinogenic areca nut, and the increase in the prevalence of sexually transmitted human papillomavirus (HPV) [2,9]. High-risk HPV, especially type 16, is predominantly associated with oropharyngeal cancer sites and is a favorable survival prognostic factor; nonetheless, global five-year survival rates average 50% [1]. Survival rates vary depending on cancer advancement, reaching 86.6% for early-stage I and II cancers. However, these rates decrease to 25–30% in stage IV cancers with greater local advancement, increased depth of invasion (DOI), node-positive (N) disease (especially N2 and N3 features), extranodal extension (ENE), and distant metastatic disease (M) [2,6].

Due to its specific localization, HNC may make it difficult for the patient to maintain an adequate nutritional status. Studies have shown that weight loss is strongly correlated with the tumor size [10]. Food intake can be impaired by functional limitations in chewing and swallowing. However, the majority of patients, even with locally advanced tumors, present with normal or higher BMI ranges at admission to the hospital. Still, many of them need nutritional support postoperatively [10,11,12,13,14,15]. It is also suggested that regardless of the overall individual body weight, up to 35% of HNC patients might be malnourished, according to laboratory and anthropometric findings. This is primarily connected to tumor-related dysphagia, and secondarily to cancer-induced cachexia, impairing normal metabolic homeostasis, causing muscle depletion, and a characteristic radiological finding of low muscle attenuation with or without loss of fat tissue [16,17].

HNC treatment may lead to several factors causing functional impairment of the stomatognathic system, such as pain, restriction of tongue mobility, impaired mouth opening due to limited mandible movement, tooth loss (loss of more than 10 teeth results in a major negative effect), concomitant mucositis, dryness of the mouth, and an impaired sense of taste during radiotherapy. Moreover, these factors may require the introduction of mashed or liquid foods, indicating and closely correlating with impairments in chewing or swallowing, which is an essential cause of a patient’s weight loss [10,12,13,18]. Malnutrition negatively impacts the quality of life and individual self-assessment of recovery chances [10].

This comprehensive review aims to determine the validity of using BMI as an indicator of potential complications in free flap reconstructive surgery following HNC, identify early and late post-surgical body weight changes, summarize evidence on complications, weight dynamics, metabolic factors, and nutritional interventions to support best practices in perioperative care.

## 2. Methods

This review included full-length English-language articles published in the range of 1989–2024 that included adult (≥18 years) HNC patients undergoing microvascular free flap reconstruction and reported nutritional, anthropometric, or metabolic variables. Preference was given to systematic reviews, meta-analyses, and large-sample original research. Older studies were included if they contributed significantly to the topic. Three electronic databases, PubMed and Google Scholar, and Web of Science (last updated 12 May 2025), were searched using combinations of the following keywords: (“head and neck cancer” OR “oral cavity cancer” OR “oropharyngeal cancer”) AND (“free flap” OR “microvascular reconstruction” OR “free tissue transfer”) AND (“body mass index” OR “BMI” OR “malnutrition” OR “obesity” OR “sarcopenia” OR “cachexia” OR “albumin” OR “prealbumin” OR “enteral nutrition” OR “nasogastric tube” OR “percutaneous endoscopic gastrostomy” OR “immunonutrition” OR “hyperglycemia” OR “enhanced recovery” OR “cancer incidence and mortality”).

Additional terms (ischemia-modified albumin, stress hyperglycemia, and sarcopenic obesity) were added iteratively, and the reference lists of selected papers were manually reviewed to identify further relevant studies. Articles were screened by the first author; uncertainties were discussed with coauthors.

## 3. Microsurgical Free Flap Reconstruction in Head and Neck Cancer

Microsurgical free flap reconstruction is considered the cost-effective, gold-standard treatment option after extended neoplasm excisions in the head and neck region. This technique has been used since the 1970s to provide patients with optimal post-surgical functional recovery and aesthetic outcomes [14,15,19].

After carefully dissecting the pedicle, a free flap is harvested from the donor site and transplanted to the recipient site by microvascular anastomoses. Depending on the included soft-tissue components, free flaps may be characterized as skin, fascial, muscular, musculocutaneous, fasciocutaneous, composite vascularized osseous, or osseomyocutaneous free flaps. The radial forearm free flap (RFFF), anterolateral thigh (ALT) flap, fibula free flap (FFF), and deep circumflex iliac artery (DCIA) flap are the most common microvascular reconstructive options for the head and neck region. The success rate for free flap transfers is about 95%, with the highest success rates described using RFFF, approaching 98.9% to 99.3% [20,21,22].

## 4. Nutritional and Metabolic Factors

BMI is a basic measurement in the primary care setting and upon admission to the hospital. While it may not differentiate fat or muscle mass deposition, it is easily accessible with clearly defined normal ranges, making it appealing for wide use in assessing a patient’s nutritional status.

Wound healing involves several overlapping phases: hemostasis, inflammation, proliferation, and remodeling. Each phase is regulated by a cascade of cellular and molecular events, including cytokine release (e.g., interleukins (IL) IL-1, IL-6, and tumor necrosis factor-alpha [TNF-α]), cell proliferation, extracellular matrix deposition (e.g., matrix metalloproteinases [MMPs]), and angiogenesis (e.g., transforming growth factor-beta [TGF-β], platelet-derived growth factor [PDGF], and vascular endothelial growth factor [VEGF]) [23]. Understanding these molecular insights and specific nutrient supplementations can help tailor interventions to optimize wound healing across different BMI categories [24].

Albumin and prealbumin are often used as markers of the patient’s nutritional status. It was found that higher preoperative albumin values correlate with decreased complication rates, especially for major wound infections, and lower mortality rates in HNC patients [14,16,25].

Albumin is a protein vital for maintaining colloidal osmotic pressure, regulating tissue perfusion, and transporting hormones, vitamins, and drugs. It has antioxidant properties and can modulate inflammatory responses, especially during the initial phases of wound healing. Low albumin levels reduce oncotic pressure, potentially leading to fluid shifts, edema, and impaired nutrient delivery to the wound site [26].

Prealbumin is a transport protein for thyroid hormones and retinol-binding protein and is a sensitive marker for nutritional status. It is currently preferred over albumin to control post-surgical nutritional status due to its shorter half-life (2–3 vs. 17–20 days). Prealbumin levels correlate with the body’s protein synthesis capacity.

It is advised to consider preoperative nutritional interventions to boost albumin levels if the serum albumin is lower than 3.0 g/dL, even at the expense of delaying surgical treatment [12,25]. A study conducted by Tsai et al. demonstrated that postoperative serum albumin levels below 3.5 g/dL were significantly associated with an increased likelihood of major wound infections in HNC patients [14]. There is strong evidence that, among other factors, a large perioperative decrease in albumin level can cause postoperative delirium (POD) [27].

Despite the recognized importance of albumin levels as a marker for nutritional status and surgical outcomes, there are very limited data specifically addressing perioperative albumin changes in HNC resection and reconstruction. In the study by Lim et al., 5.0% of patients exhibited serum albumin levels below 3.5 g/dL before surgery; this proportion increased to 13.3% after two months [28].

The study by Kao et al. examined changes in serum albumin levels during the perioperative period in HNC patients with liver cirrhosis, correlating these changes with surgical outcomes. Preoperative albumin levels ranged from 1.9 to 5.2 g/dL (median 3.9 g/dL), and on the first postoperative day, levels ranged from 1.6 to 3.5 g/dL (median 2.7 g/dL). The percentage drop in albumin levels was significant: 27.1% in patients with preoperative levels > 3.5 g/dL and 32.7% in those with levels ≤ 3.5 g/dL. Moreover, greater intraoperative blood loss was associated with larger drops in albumin levels [29].

BMI is considered a crude measurement; hence, the literature suggests using a modified nutrition-related index (NRI) to simplify preoperative malnutrition screenings. NRI is calculated using the following formula: NRI = [1.519 × serum albumin (g/L)] + [41.7 × (current weight/ideal body weight (IBW) (kg)]. IBW is usually calculated using the Devine formula for men and the Robinson formula for women [30]. Patients with an NRI of less than 97.5 are considered moderately malnourished, and those with scores less than 83.5 are considered severely malnourished [31].

Electrolyte abnormalities are commonly encountered in HNC patients, most frequently manifested as hypophosphatemia and hypocalcemia. Specific supplementation should be considered if needed [32].

Ischemia-modified albumin (IMA) is a marker of oxidative stress and ischemia. In HNC, elevated IMA levels indicate increased oxidative stress due to the tumor’s metabolic demands and inflammatory response. The study by Madhumita et al. focused on IMA as a marker of oxidative stress in patients with locally advanced HNC. Their findings suggested that IMA, particularly albumin-adjusted IMA (AdjIMA), reflects oxidative stress levels in these patients before the initiation of cancer therapy [33]. Few studies suggest that IMA levels rise in response to ischemic events during surgeries, indicating its potential as a marker; however, the evidence is not yet conclusive or universally accepted.

High levels of blood glucose (BG), especially in patients with diabetes, can heighten the chances of complications occurring during and after surgery. Research has shown that an HbA1c level exceeding 8% independently increases the risk of wound complications [34]. Elevated BG levels impair immune function and promote inflammation, potentially worsening recovery outcomes.

Serum insulin-like growth factor-I (IGF-I) levels could potentially serve as a biomarker to monitor the progress of wound healing after surgery. Higher levels of IGF-I might indicate more effective healing processes due to its role in promoting keratinocyte migration, epithelialization, and wound bed contraction. Lower serum IGF-I levels could help identify patients at risk of delayed healing, particularly in elderly patients or those with chronic conditions [35,36]. However, specific studies examining the role of IGF-I in the context of HNC reconstruction surgery are lacking.

## 5. Impact of Obesity on Head and Neck Cancer Surgery with Free Flap Reconstruction

Deng et al. and Ringel et al. noted that obesity has a multifactorial impact on antitumor immunity due to its effects on immune-metabolic changes, systemic metabolic dysregulation, and the production of adipose-derived cells and proinflammatory factors that promote tumor growth and metastasis [37,38].

A retrospective study by Asaad et al., including 4000 free flap surgeries, concluded that a higher BMI range prolonged surgical times, yet no severe complications were noted [39]. Joshi et al. confirmed these findings; however, they highlighted that obesity is a systemic issue and may be associated with complex multimorbidity. The presence of significant factors such as hypertension, diabetes, and chronic obstructive pulmonary disease, in addition to advanced age and the tendency for tobacco addiction in the typical HNC patient, makes postoperative care challenging [11,40,41,42].

Furthermore, none of the above studies extract the average intraoperative ischemia time of the free flap from the total surgery time. A study by Iamaguchi et al. suggested that obese patients are at risk of prolonged surgical times for microvascular anastomosis in FFF [43]. Although intraoperative ischemia time is one of the factors that correlate with free flap loss, several factors play more significant roles, such as surgical technique, surgeon experience and expertise, the volume of free flap reconstructions performed at a particular center, and various patient factors [44].

Matošević et al. conducted an important study on 113 patients undergoing free flap reconstruction due to oral and oropharyngeal cancers collected over 5 years in one tertiary university referral center. The authors noted that prolonged surgery time was associated with flap failure and mortality [45]. This indirectly suggests that performing fewer procedures (mean 22.6 yearly) predisposes patients to a higher complication rate and highlights the need to develop HNC-devoted oncological centers [16].

The decision about the selection of the donor site and, consequently, the type of flap, is based on several factors, such as local anatomy, type and dimensions of the defect, donor site morbidity, surgeon expertise, patient’s socioeconomic status, and many others [46]. Body weight directly correlates with the thickness of the flap, especially in the case of the ALT flap [47]. While thick and bulky flaps can sometimes lead to poor aesthetic and functional outcomes in the anatomically complex head and neck region, these characteristics are not always disadvantages in head and neck surgery. In certain cases, thickness and bulkiness are desirable, as they can address functional issues, such as reconstructing a base of the tongue defect, and aesthetic concerns following procedures like an extended radical neck dissection. Although the literature proposes several techniques for flap thinning, the extensive reduction in flap mass can jeopardize partial or total flap survival. According to Agostini et al., the overall incidence of vascular-related complications after flap thinning is significant, reaching up to 13.4% [48].

Higher BMI correlates with increased intraoperative blood loss, primarily attributed to the heightened vascularity of adipose tissue and technical challenges, including prolonged surgical times, encountered in obese patients [39].

Delayed extubation up to 24–48 h after free flap reconstruction in the head and neck is a safe alternative to primary tracheostomy for most patients and should be considered selectively for patients with specific risk factors [49]. Elective tracheostomy is associated with higher rates of postoperative complications, including increased incidences of dysphagia, pneumonia, and prolonged hospital stays, compared to strategies aligned with the Enhanced Recovery After Surgery (ERAS) protocol. Additionally, higher BMI contributes to an increased likelihood of requiring tracheostomy due to issues such as airway obstruction from excess neck tissue, respiratory compromise, difficulties with intubation, and potential complications related to prolonged ventilation [50].

While obesity generally increases the risk of complications in surgery, including infections, according to the literature, it does not seem to play a significant role in free flap surgery [11,42].

Perioperative hypothermia during free flap surgery, particularly in HNC resections, significantly increases the risk of arterial thrombosis and total flap loss, resulting in longer hospital stays. Moellhoff et al. found that patients with hypothermia (<36.0 °C) had higher rates of total flap loss (6.6% vs. 3.0%) and arterial thrombosis (4.6% vs. 1.9%) compared to normothermic patients (≥36.0 °C). Hypothermia is associated with increased postoperative intensive care unit (ICU) admissions and longer postoperative hospital stays. Due to the body’s physiology and the thermal properties of adipose tissue, obesity provides a degree of protection against perioperative hypothermia compared to underweight patients [51,52]. Maintaining normothermia (36–36.5 °C) with active warming strategies and continuous temperature monitoring is crucial to optimize surgical outcomes and patient recovery. To provide a clearer overview, Table 1 summarizes the impact of obesity on head and neck surgery outcomes.

## 6. Weight Dynamics

Crippen et al. suggested that a BMI under 18.5 and a recent weight loss of 10% in the 6 months before surgery increase the perioperative mortality risk by over four-fold, simultaneously suggesting a reduced ability to tolerate head and neck free flap reconstructive surgeries. These findings may be partially explained by cardiac dysfunction induced by the inflammatory cytokines that promote cachexia; hence, many authors suggest that obesity may have a protective value against severe cardiac events or post-surgical mortality [41,54,55]. Several authors report that overweight and early-stage obese patients tolerate combined therapies (surgery, radio-, and chemotherapy) better than normal BMI or underweight patients, due to the nutrient reserve [56]. However, the benefits of obesity in this context may be overstated without taking into account the potential negative impact of sarcopenic obesity on surgical recovery and overall health [57].

Many publications highlight weight loss in the long-term postoperative period of HNC treatments (more than 6 months), mainly due to radio- and chemotherapy, tooth loss, depression, and psychological variables. However, limited research exists with reliable quantitative data about body weight alterations due to the free flap reconstruction of the head and neck region [10,30,58,59]. Karnell found that HNC patients with weight changes, especially weight loss of more than 5% by the three-month post-diagnosis period, significantly lowered the 5-year survival rates compared to patients with a stable weight [60].

Trismus is an important but often overlooked nutritional and metabolic stressor following HNC free flap reconstruction. A meta-analysis involving 2786 patients reported that approximately 17% of HNC patients fall below the ≤35 mm trismus threshold before any intervention, increasing to 44% at six months and remaining above 30% in the long term, with the highest incidence when ablative surgery is followed by adjuvant chemoradiotherapy [61]. In comparison, in a prospective chemoradiation study, trismus emerged as early as week 3 of treatment, peaked at 42% during week 6, and showed a strong correlation with radiation doses exceeding approximately 37 Gy to the ipsilateral lateral pterygoid. A significant 90% of these patients also necessitated prophylactic gastrostomy for adequate caloric intake [62]. Limited jaw mobility in this context hinders oral feeding and complicates dental care. Thus, perioperative protocols for free flap patients should include screening for mouth opening preoperatively, commencing jaw-mobilizing exercises and patient education before or at the start of adjuvant therapy, and incorporating dietitian-led enteral support when radiation exposure to the masticatory system is unavoidable. Currently, there is insufficient reliable data comparing trismus outcomes between surgery-first and radiotherapy-first approaches; critical elements such as dose-effect thresholds, ideal rehabilitation timing, and accurate long-term recovery patterns remain poorly understood.

## 7. Perioperative Nutritional Interventions

Preoperative carbohydrate drinks administered 2 h before surgery are widely used in clinical settings, supported by evidence showing benefits such as muscle preservation and reduced metabolic stress during the perioperative period, in line with ERAS protocols [63]. However, there is no definitive consensus on their impact on reducing postoperative complications, and there may be conflicting findings. Mitigating insulin resistance, stabilizing BG levels, and supporting metabolic balance throughout surgery may also be beneficial in postoperative HNC reconstruction surgery [64,65]. Their application in preventing postoperative complications remains a topic for ongoing research and debate within the medical community.

Conservatively adopted HNC post-surgical recommendations of “nil by mouth (NBM)” for 6–12 days post-surgery before the reinitiation of oral food intake require nasogastric (NG) tube or percutaneous endoscopic gastrostomy (PEG) feedings as the two principal post-surgical feeding strategies for HNC patients unable to masticate and swallow. Studies note that early postoperative tube feeding within 24 h is safe and recommended; ERAS protocols advocate for commencement even within 12 h [12,63,66]. The European Society for Parenteral and Enteral Nutrition (ESPEN) guidelines note that an NG tube is the preferred method over PEG in the post-surgical period; however, other studies note that definitive conclusions cannot be drawn because of the heterogeneity of the HNC group and many variables needing to be taken into account, such as multimodal treatment options, possible adverse events, patients’ quality of life, and social aspects of both feeding methods [66,67].

The main purpose of enteral feeding is to maintain or improve the patient’s nutritional status, improve post-surgical oral hygiene, and reduce complications to prevent further weight loss [68]. ERAS guidelines for head and neck procedures discourage early resumption of oral intake, emphasizing patient safety and recovery, particularly due to surgical complexities and associated risks [69]. To the best of our knowledge, only a few recent papers provide surgeons with adequate guidelines about the timing of introducing oral feeding. Kerawala et al., contrary to the conservative approach, claim that the early (“to start fluids and soft diet on the day following surgery”) introduction of oral nutrition supports the maintenance of proper oral hygiene due to increased saliva production and mechanical exfoliation of the epithelium during movement [67,70,71]. PEG is advised if the need for long-term nutritional intervention is anticipated; however, in the group with locally advanced HNC, PEG site metastases may occur as a rare complication (1–2%) [72]. Still, careful consideration of advantages and disadvantages is required when selecting the method of enteral nutrition intervention and its timing. Moreover, Gellrich et al. found that despite providing the patient with the long-term possibility of proper nutrition using PEG, 61% still experienced weight loss at the 6-month follow-up, which is equal to the result in a group requiring a liquid diet [10].

Postoperative feeding strategies are critical for recovery and can vary based on the patient’s needs and surgical outcomes. Table 2 provides a comprehensive overview of these strategies.

## 8. Management of Stress-Induced Hyperglycemia

Stress hormones released during surgery, such as cortisol and catecholamines, elevate hepatic glucose production and suppress insulin secretion, leading to elevated BG levels, particularly in obese patients. This response not only impairs immune function but also promotes inflammation, potentially worsening recovery outcomes. Hyperglycemia (>180 mg/dL) postoperatively is associated with higher risks of surgical site infections, delayed wound healing, and prolonged hospital stays. Importantly, stress-induced hyperglycemia can occur even without a prior diabetic history, highlighting the need for proactive management strategies. Subcutaneous rapid-acting insulin analogs are recommended to correct hyperglycemia during the intra- and perioperative period [65,73].

Several organizations recommend different glycemic targets. The Society for Ambulatory Anesthesia (SAMBA) suggests intraoperative BG < 180 mg/dL. The American Association of Clinical Endocrinologists (AACE) and the American Diabetes Association (ADA) target 140–180 mg/dL in critically ill patients. The Society of Critical Care Medicine (SCCM) advises treating BG ≥ 150 mg/dL with a goal of <180 mg/dL [74,75,76].

Hwang et al. conducted a pilot study about intraoperative feeding via an NG tube during free flap HNC surgery, randomly assigning two groups to receive either feeding or fasting. This study noted that severe adverse events did not differ between the groups. Nonetheless, statistically significant reductions in wound dehiscence, marginal necrosis, lower concentrations of proinflammatory plasma IL-6 and IL-8, and shortened hospital length of stays were found in favor of the fed group [77].

## 9. Other Perioperative Challenges

There is currently no definitive consensus on the optimal mean arterial pressure (MAP) in HNC free flap surgeries to ensure sufficient tissue perfusion. However, Meng et al. suggest that in noncardiac surgeries, the perioperative MAP target should be based on the patient’s baseline blood pressure (BBP). They recommend a range of 65–95 mmHg for normal BBP to minimize the risk of complications [78].

Brauer et al. found that free flap patients suffering from hypertension, which is more common in obese individuals, have a greater risk for unplanned reoperations [15].

Moreover, many patients undergoing head and neck reconstruction are at risk of intraoperative hypotension due to factors such as the use of anesthetic agents, opioid analgesics, prolonged operative times, blood loss, insensible fluid loss, hypothermia, and associated medical comorbidities [79]. A MAP below 60 mmHg for an extended period during surgery can be a significant predictor of unfavorable postoperative outcomes. Additionally, some responses to hypotension, such as administering larger volumes of crystalloids or using inotropes, may also increase the overall risk of flap failure [80,81].

A study by Roshanov et al. found that withholding ACE inhibitors or angiotensin II receptor blockers 24 h before noncardiac surgery reduced the risk of death, stroke, or myocardial injury within 30 days and decreased intraoperative hypotension [82].

Multiple studies note that the use of vasopressors during the microvascular free tissue transfer for HNC reconstruction does not increase flap failure rates or other postoperative complications. It may even enhance hemodynamic stability and reduce fluid administration, potentially decreasing postoperative issues. However, further prospective studies are necessary to develop evidence-based guidelines for vasopressor use in free flap surgery [79,83].

Blood transfusion practices in head and neck surgery, particularly during complex procedures like free flap reconstructions, have evolved significantly. Recent studies highlight that higher transfusion volumes are associated with poorer outcomes, including increased mortality and higher infection rates. Evidence supports a shift towards more conservative transfusion thresholds, such as hematocrit < 25%, compared to traditional criteria (<30%). Critical care literature suggests that transfusing at lower hemoglobin levels (e.g., <7 g/dL) may be safer and more effective in maintaining patient health post-surgery, improving outcomes, and minimizing complications [84,85,86]. A possible solution to support proper nutritional status during the recovery period after mandibulectomy involves reconstruction with an osseous or osseomyocutaneous fibula or iliac crest free flap with immediate dental implants, first described by Urken et al. in 1989 [87,88]. There is a paucity of data on dental rehabilitation involving fibula free flaps or other bony free flaps and their effects on short- and long-term post-surgical nutritional status [19,89].

Several studies found that depressive symptoms after HNC treatment are associated with a generally worse survival rate, reduced oral energy intake, and greater weight loss in long-term observation (2.5–6–12 months), especially due to mouth dryness, impaired social contact, and eating disorders, compared to no symptoms [90,91,92]. Antidepressant interventions are advised to improve treatment compliance, quality of life, and the overall survival rate [93].

**Figure 1:**
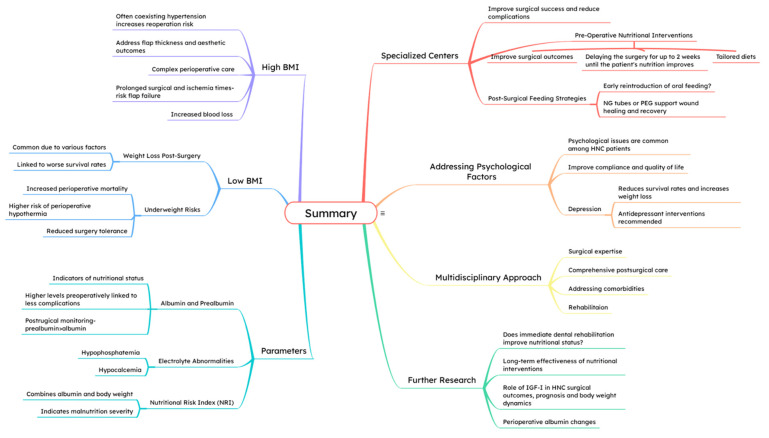
Graphical Summary.

This seems very important to consider while tailoring individual HNC treatment plans because the overall incidence of depression among HNC patients is reported to be 9.3–11.5%, depending on the population and the cancer location, with a peak of up to 28.5% in the laryngeal cancer group [93,94]. Patients undergoing HNC treatment should be evaluated individually by a multidisciplinary team for nutritional requirements. Protocols and guidelines from ESPEN [63] or the American Society for Parenteral and Enteral Nutrition (ASPEN) [95] are recommended but are rather general, only briefly addressing HNC patients [68]. Figure 1 provides a graphical summary of the article’s key findings.

The review advocates for further research in several critical areas: the role of insulin-like growth factor-I (IGF-I) in wound healing, long-term weight changes, optimal perioperative nutritional protocols, and ischemia-modified albumin (IMA) as a marker of oxidative stress and ischemia. It underscores the importance of specialized HNC centers to improve patient outcomes through expert surgical and nutritional care. Additional research in these areas will provide valuable insights and help refine guidelines for the comprehensive management of HNC patients undergoing reconstructive surgery.

## 10. Limitations

This comprehensive review has several limitations. Although we aimed to capture a wide range of high-quality evidence, the selection of studies may have been influenced by availability and accessibility. While we included systematic reviews, meta-analyses, and original studies with large sample sizes, some included articles were retrospective or based on expert opinion, which may introduce bias or limit generalizability. The heterogeneity of study designs, populations, and outcome measures prevented a formal meta-analysis. Additionally, there is limited high-quality research specifically addressing certain aspects, such as the role of IGF-I or ischemia-modified albumin in head and neck cancer surgery. Finally, while efforts were made to provide up-to-date evidence, rapidly evolving fields such as perioperative nutrition and metabolic monitoring may render some findings outdated in the near future. Further prospective, multicenter studies are needed to validate and expand upon the conclusions drawn in this review.

## 11. Conclusions

This review underscores the profound impact of perioperative nutritional status on the outcomes of HNC reconstructive surgery. It emphasizes that adequate nutritional management, both preoperatively and postoperatively, is essential for optimizing surgical success and enhancing patient recovery. Key findings highlight the importance of albumin and prealbumin as nutritional markers, the benefits of preoperative nutritional interventions, and the necessity of tailored pre-, intra-, and post-surgical feeding strategies. BMI also plays a significant role, as both high and low BMI can impact surgical outcomes.

Furthermore, the review highlights the necessity of a multidisciplinary approach to optimizing nutritional status, as both high and low BMI can impact surgical outcomes, and effective management requires coordination among various healthcare professionals.

## Figures and Tables

**Table 1:** Impact of Obesity on Head and Neck Surgery.

Aspect	Description	Impact on Surgical Outcomes	Notes/Recommendations	GoE	SoR
Immune and Metabolic Responses	Obesity affects immune-metabolic changes, systemic metabolic dysregulation, and the production of adipose-derived cells and proinflammatory factors.	Promotes tumor growth and metastasis, and affects antitumor immunity.	Consider preoperative optimization and careful monitoring.	II	B
Surgical Times	Higher BMI can prolong surgical times due to technical challenges and increased tissue manipulation.	Prolonged surgery increases the risk of complications, including infection and blood loss.	Minimize surgery duration through preoperative planning.	II	B
Intraoperative Free Flap Ischemia Time	Time can be prolonged in obese patients, although several factors, like surgical technique and surgeon experience, play a more significant role in free flap loss.	Prolonged ischemia time correlates with an increased risk of free flap loss; however, other factors are more critical.	Optimize surgical techniques to reduce ischemia time; focus on improving surgical expertise and center experience.	III	B
Donor Site Selection and Flap Thickness	Obesity directly correlates with increased flap thickness, especially in ALT flaps. Thickness and bulkiness are sometimes desired to solve functional and aesthetic issues.	Thick and bulky flaps can lead to poor aesthetic and functional outcomes, but may be beneficial in certain cases.	Consider techniques for flap thinning when necessary; however, be cautious of compromising vascular integrity. The selection of donor sites should be based on defect requirements.	III	B
Intraoperative Blood Loss	Higher vascularity of adipose tissue increases intraoperative blood loss.	Increased blood loss can complicate surgery and prolong recovery.	Utilize strategies to minimize blood loss, such as meticulous hemostasis and careful tissue handling.	III	B
Airway Management and Extubation	Obesity increases the likelihood of requiring a tracheostomy due to airway obstruction and respiratory compromise.	Delaying extubation can reduce complications compared to elective tracheostomy.	Individualize the airway management plan based on the extent of surgery, patient’s BMI, and comorbidities. Consider delaying extubation when appropriate.	III	B
Hypothermia	Obesity provides some protection against perioperative hypothermia.	Maintaining normothermia is crucial for optimizing outcomes and recovery.	Implement active warming strategies and continuous temperature monitoring.	II	A
Weight Loss and Perioperative Mortality Risk	Obesity may provide protective nutrient reserves, whereas low BMI is associated with higher perioperative mortality risk.	Underweight patients have a reduced ability to tolerate extensive surgeries and a higher risk of complications.	Ensure nutritional optimization pre- and post-surgery for all BMI categories.	II	A

GoE—Grade of Evidence: I—Meta-analysis or ≥1 high-quality RCT, II—Prospective cohort or lower-quality RCT, III—Retrospective or case-control study, IV—Case series, V—Expert opinion. SoR—Strength of Recommendation: A—Strong, B—Moderate, C—Weak [53].

**Table 2:** Pre- and Post-Surgical Feeding Strategies.

Feeding Strategy	Description	Advantages	Disadvantages	Notes/Recommendations	GoE	SoR
Preoperative Carbohydrate Drinks	Administered 2 h before surgery.	Muscle preservation, reduced metabolic stress, and stabilization of blood glucose levels.	Impact on reducing postoperative complications not definitively proven.	Supported by evidence in ERAS protocols for overall benefits.	II	B
NG Tube	Tube feeding through the nose to the stomach.	Preferred method post-surgery, supports early feeding within 12–24 h.	May cause discomfort, intended for short-term use.	ESPEN guidelines note that an NG tube is preferred over PEG in the post-surgical period.	II	A
PEG	Tube feeding directly into the stomach through the abdominal wall.	Suitable for long-term feeding needs, maintains nutritional status.	Risk of site infection, risk of metastases (1–2%), may still result in weight loss.	PEG is advised if long-term nutritional intervention is anticipated. Careful consideration is required for advanced HNC cases.	III	B
Early Oral Feeding	Introducing fluids and a soft diet shortly after surgery.	Supports oral hygiene due to increased saliva production and mechanical exfoliation of the epithelium; may improve patient comfort.	Risk of aspiration, needs careful monitoring; a conservative approach often delays the reintroduction of oral intake	ERAS protocols discourage early resumption of oral intake	II	B
NBM	No oral intake for 6–12 days post-surgery.	Allows healing of surgical sites without the stress of swallowing.	Requires NG tube or PEG feeding for nutrition, potential for patient discomfort, prolonged recovery.	A common conservative approach, used to avoid complications during the initial healing phase.	III	C

GoE—Grade of Evidence: I—Meta-analysis or ≥1 high-quality RCT, II—Prospective cohort or lower-quality RCT, III—Retrospective or case-control study, IV—Case series, V—Expert opinion. SoR—Strength of Recommendation: A—Strong, B—Moderate, C—Weak [53].

## Data Availability

Not applicable.

## Abbreviations

AACE	American Association of Clinical Endocrinologists
ACE	Angiotensin-Converting Enzyme
ADA	American Diabetes Association
ALT	Anterolateral Thigh
ASPEN	American Society for Parenteral and Enteral Nutrition
BBP	Baseline Blood Pressure
BG	Blood Glucose
BMI	Body Mass Index
DCIA	Deep Circumflex Iliac Artery
DOI	Depth of Invasion
ENE	Extranodal Extension
ERAS	Enhanced Recovery After Surgery
ESPEN	European Society for Parenteral and Enteral Nutrition
FFF	Fibula Free Flap
HNC	Head and Neck Cancer
HPV	Human Papillomavirus
IBW	Ideal Body Weight
ICD	International Classification of Diseases
ICU	Intensive Care Unit
IGF-I	Insulin-Like Growth Factor-I
IMA	Ischemia-Modified Albumin
MAP	Mean Arterial Pressure
MMPs	Matrix Metalloproteinases
NBM	“Nil by Mouth”
NCCN	National Comprehensive Cancer Network
NG	Nasogastric
NRI	Nutrition-Related Index
PEG	Percutaneous Endoscopic Gastrostomy
POD	Postoperative Delirium
RFFF	Radial Forearm Free Flap
SAMBA	Society for Ambulatory Anesthesia
SCCM	Society of Critical Care Medicine
